# Compliance with Standard Precautions among Clinical Nurses: Validity and Reliability of the Italian Version of the Compliance with Standard Precautions Scale (CSPS-It)

**DOI:** 10.3390/ijerph16010121

**Published:** 2019-01-04

**Authors:** Daniele Donati, Valentina Biagioli, Claudia Cianfrocca, Maria Grazia De Marinis, Daniela Tartaglini

**Affiliations:** 1 Department of Biomedicine and Prevention, Tor Vergata University of Rome, 00133 Rome, Italy; valentina.biagioli@pec.ipasvi.roma.it (V.B.); c.cianfrocca@unicampus.it (C.C.); 2 Research Unit Nursing Science, Campus Bio-Medico University of Rome, 00128 Rome, Italy; m.demarinis@unicampus.it (M.G.D.M.); d.tartaglini@unicampus.it (D.T.)

**Keywords:** standard precautions, guideline compliance, infection control, instrument validation, nursing

## Abstract

Background: The compliance with Standard Precautions (SPs) guidelines, as a primary strategy for the prevention of healthcare associated infections, is still suboptimal among healthcare providers. However, no instrument measuring nurses’ compliance with SPs is available in Italian. This study aims to assess the validity and reliability of the Compliance with Standard Precaution Scale–Italian version (CSPS-It) among clinical nurses. Methods: The study consisted of two phases: (1) translation and cross-cultural adaptation of the CSPS; (2) validity and reliability evaluation of the CSPS-It. Confirmatory factor analysis (CFA) and hypothesis testing were performed to evaluate the construct validity. Cronbach’s alpha, intra-class correlation coefficient of test-retest scores, and item-total correlations were computed to establish reliability. Results: The CSPS-It showed a sound validity and reliability. The unidimensional model tested at CFA yielded acceptable fit indices. The hypothesis testing supported better nurses’ compliance based on participation in at least one training course on SPs. Conclusions: The CSPS-It is a valid and reliable instrument for measuring the compliance with SPs among clinical nurses. This version will allow for the conduction of further studies in favor of progress in this specific field of research. Managers should pay greater attention in monitoring compliance with SPs among clinical nurses.

## 1. Introduction

Healthcare-associated infections (HAIs) can be considered the most frequent adverse event that occurs in providing healthcare worldwide [1,2]. It has been estimated that over 4 million patients in Europe and 1.7 million in the United States develop a HAI every year, with a higher prevalence in developing countries [1,3]. HAIs are associated with longer hospital stays, increased mortality, increased healthcare costs, and psychosocial and economic burdens on the individuals involved, as well as on their families and communities [1,4].

The strict implementation of standard precautions (SPs) is the primary strategy for the prevention of HAIs both in healthcare professionals and in patients [5]. SPs represent the most complete and recent guidelines for the prevention of infectious risk [6,7,8,9]. SPs are based on the principle that all blood, body fluids, secretions, excretions (except sweat), nonintact skin, and mucous membranes may contain transmissible infectious agents [5,7].

SPs guidelines include appropriate hand hygiene, use of gloves and other personal protective equipment (PPE), appropriate cleaning and disinfection of patient care equipment and environment surfaces, right waste disposal, correct management of used needles and other sharp objects, and appropriate cough etiquette [5,6,7,10]. These measures are essential for effective infection control because they disrupt the spread of microorganisms from one patient to another via healthcare workers’ hands or uniforms, reduce exposure of healthcare workers to infectious agents, and reduce environmental contamination [5,6,7]. SPs should be applied during every healthcare activity for all patients at all times to prevent HAIs among patients and healthcare professionals, regardless of the infectious agents’ known or presumed status [5,6,7].

Although health organizations worldwide recognize SPs as the best way to prevent HAIs [11], the compliance with these measures is still suboptimal among healthcare providers [12,13,14,15,16]. In particular, compliance with SPs should be enhanced among nurses, who are involved in direct and repeated patient care [17] and thereby can be more exposed to microorganisms associated with cross-infections [15]. Increasing evidence also indicates that nurse compliance with SPs can contribute to the reduction of HAIs among patients and healthcare providers, improving the effectiveness and safety of the care provided [5,18,19].

The compliance rate is defined as the extent to which the behaviours of a worker coincide with the prescriptions of the authority [20]. Thus, such infection control and prevention behaviours should be carefully monitored among nursing staff to guarantee the maintenance of optimal levels of safety. For this reason, a valid and reliable tool to measure nurses’ compliance with SPs is required.

At least 18 instruments have been developed to investigate the level of nurses’ compliance with SPs [21]. Among the available tools, the Compliance with Standard Precautions Scale (CSPS) appears to be the most used and validated self-report scale based on the concept of SPs, as an update of the universal precautions concept used in previous studies [22,23,24]. The CSPS measures the level of self-reported compliance with SPs among nurses and nursing students during their daily clinical practice [24]. 

Several scholars translated and validated the CSPS in multiple languages to assess the compliance with SPs [25,26,27]. In fact, the CSPS is the only instrument for which cross-cultural pilot testing has been carried out, involving 19 experts from 16 different countries [9]. Thus, the CSPS is considered globally applicable to both developed and developing countries [9].

To the extent of our knowledge, no instrument measuring compliance with SPs is available in Italian. In order to contribute to a more complete and accurate assessment of the compliance with SPs among Italian nurses, it is necessary to find a valid and reliable tool. Thus, the aim of this study was to translate the CSPS into Italian and to examine the validity and reliability of the Italian version of the scale (CSPS-It) among clinical nurses.

## 2. Materials and Methods

### 2.1. Study Design and Setting

The present study was conducted at an Italian university hospital in Rome, Italy from February 2017 to July 2018. The hospital has about 280 beds and is funded by the National Health System.

**Figure 1:**
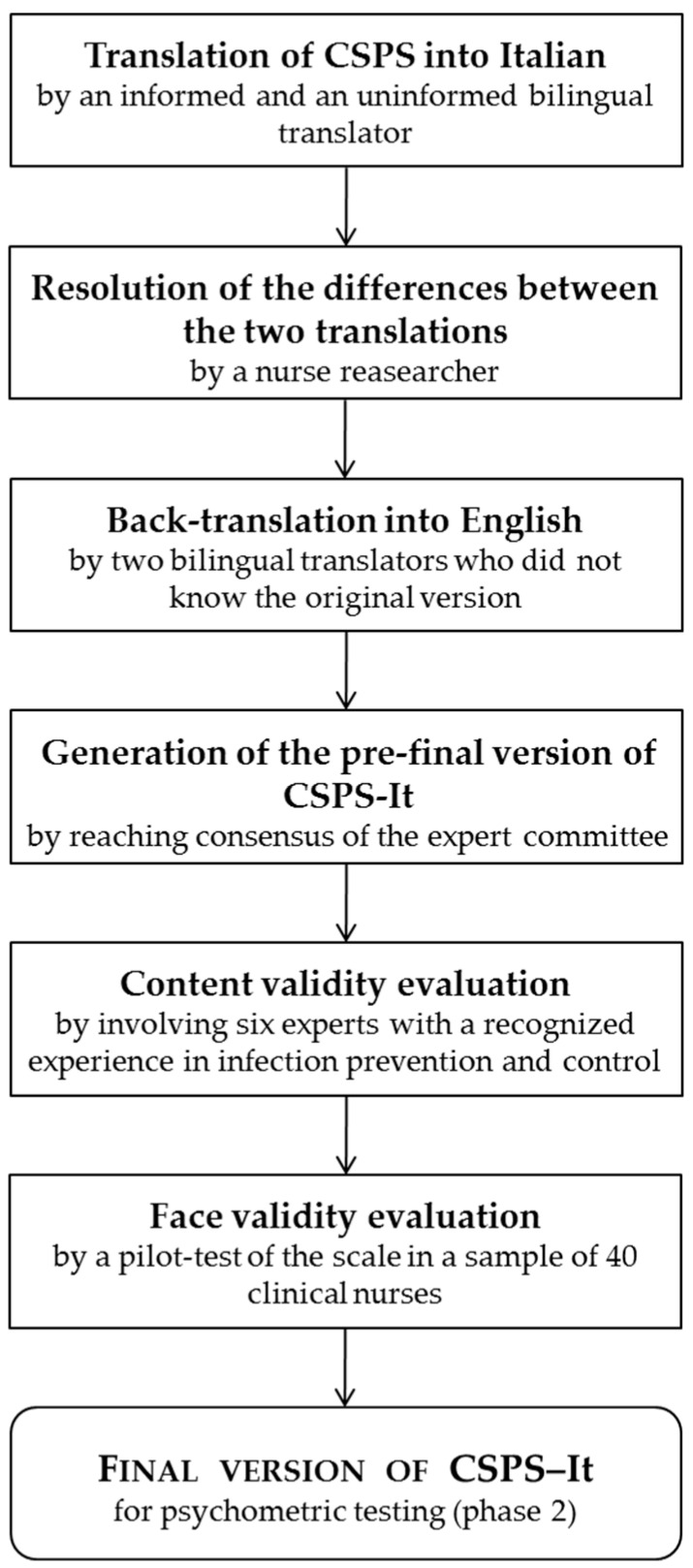
Methodological steps of phase 1.

The study consisted of two phases:

(1) Translation into Italian and cross-cultural adaptation of the CSPS (Figure 1); and
(2) Validity and reliability evaluation of the CSPS–Italian version (CSPS-It).

Phase 1 was conducted from February 2017 to February 2018 and phase 2 was conducted from March 2018 to July 2018. 

### 2.2. Ethical Considerations

The healthcare directorate of the hospital where the study was conducted approved this research. The permission to translate and use the CSPS was granted by the original author of the scale (approval code: CO500D24-201702). Taking part in the study was voluntary and data were anonymously collected to improve participation. Informed consent about the study aim and procedures, as well as information about the anonymity of the collection of data collection were included in the survey. Participant’s consent was obtained through a specific form to complete in the online survey and it was a precondition to participate in the study.

### 2.3. Instruments

The CSPS is a self-administered questionnaire composed of 20 items. The 20 items describe the use of protective devices, disposal of sharp instruments and waste, decontamination of spills and used articles, and prevention of cross-infection [24]. CSPS satisfactory concurrent and construct validity showed in previous studies [9,24]. The reliability of the CSPS was mainly evaluated through Cronbach’s alpha and by comparing two different groups: nurses and nursing students [24]. The internal consistency of the original version was acceptable (Cronbach’s alpha was 0.73) and the CSPS reliability was high (intra-class correlation coefficients were 0.79 and 0.74 for the two-week and three-month test-retest) [9,24]. Participants were asked to indicate the extent to which they believed themselves to be compliant with several SPs behaviours on a four-point Likert scale ranging from 1 (never) to 4 (always). The CSPS includes both positively and negatively (items 2, 4, 6, and 15) worded statements. To compute the total score, item scores were summed together: only the maximum compliance option (“always” for positive items and “never” for negative items) was scored 1, while the other options were scored 0. This is because nurses are expected to fully comply with local SPs guidelines [20]. Thus, the total score can range from 0 to 20, with higher values indicating a better compliance with SPs. In addition, it was possible to calculate the average compliance rate for each item, which is the percentage of maximum compliance among all participants.

### 2.4. Phase 1

#### 2.4.1. Translation and Cross-Cultural Adaptation Process

Translation and cross-cultural adaptation of the CSPS followed the guidelines for the cross-cultural adaptation and translation of self-reported instruments [28]. A scheme of the entire process is reported in Figure 1. Initial translation in Italian was conducted by two independent bilingual translators with Italian as their primary language (an infection control expert nurse working in a university hospital and a professional translator without knowledge of SPs). Both translators were able to detect ambiguous items (e.g., item 13) and provided a written report of the translation process. Any difference between the two translations was resolved by a third nurse researcher. The Italian version of the CSPS was then independently back-translated into English by two different bilingual translators, with English as their primary language. They were masked to the original English version of the questionnaire. A group of experts (the project leader, who was a nurse researcher, a methodology expert, the involved professional translators, a doctor specialized in infectious diseases and public health and an infection control nurse) developed the pre-final version of the questionnaire for field testing. All experts had a good fluency in both English and Italian languages. The experts reviewed all the translations and reached a consensus on any discrepancy achieving semantic, idiomatic, experiential, and conceptual equivalence between the original version of the CSPS and the Italian version.

#### 2.4.2. Content Validity Evaluation

In order to establish the content validity of the CSPS-It, six experts were involved in the item evaluation process (the project leader, two infection control nurses, a doctor specialized in infectious diseases and public health, a doctor specialized in preventive medicine, and a nurse researcher). The experts had a recognized experience in infection prevention and control. They were asked to rate each item for its relevance to the SPs using a four-point Likert scale ranging from 1 (not relevant) to 4 (highly relevant) [29]. Content validity was then established by calculating the item-level content validity index (I-CVI) and the scale-level content validity index (S-CVI/Ave). An I-CVI ≥ 0.78 for 6 to 10 members [30] and a S-CVI/Ave ≥ 0.90 were considered acceptable [29].

#### 2.4.3. Face Validity Evaluation

The prefinal translation of the CSPS-It was then pilot-tested in a convenience sample of 40 clinical nurses selected among those working in the same university hospital as the others of phase two, and who met the same inclusion criteria. The concept of SPs was made clear. Participants were asked to complete the CSPS-It and to provide their comments primarily with regard to comprehensibility and applicability to the Italian context in order to assess the face validity of the CSPS-It.

### 2.5. Phase 2

#### 2.5.1. Participants

Participants for the psychometric evaluation of the CSPS-It were nurses working in a hospital that adopted SPs as infection control guidelines. Inclusion criteria required participants to be providing direct care for patients across several clinical units of the hospital, and to have an institutional email address. Head nurses, nurses working in administrative areas or in other non-clinical contexts, and other healthcare providers were excluded because they are usually less involved in direct clinical care and thereby they are not part of the target population. 

#### 2.5.2. Data Collection

Data were collected during March 2018. Participants were asked to complete an online questionnaire through an institutional nursing web survey. Socio-demographic characteristics (sex, age, education, clinical experience, clinical setting, participation in at least one training course on SPs) had been collected in a specific section before the questionnaire. An informatics platform was used to emphasize a confidential approach and allow participants to complete the survey anywhere using computers, smartphones, or tablets.

#### 2.5.3. Statistical Analysis

Streiner and Norman criteria was used to calculate the minimum sample size [31]. We have estimated a sample of 10 participants for each item. Given that the CSPS-It included 20 items, we have estimated 200 participants as minimum sample size to perform the factor analysis. In order to be representative of all staff nurses working within the university hospital, we invited all clinical nurses to participate in the phase 2, with the exception of the 40 nurses involved in the face validity (n = 308). In addition, we considered that the participants’ response rate could be lower than 70%.

Descriptive statistics (means, standard deviations [SD], frequencies, and percentages) were calculated for all study variables. In line with a comprehensive concept of SPs [9,24], a unidimensional model of the CSPS-It was tested through confirmatory factor analysis (CFA). Since the scale includes ordinal items, CFA was performed using the weighted least squares means and variance adjusted estimator (WLSMV) [32]. To evaluate the fit of the model, the following fit indices and values of good fit were considered: Chi-square (χ^2^): Not significant; the comparative fit index (CFI): > 0.90; the Tucker-Lewis index (TLI): > 0.90; the root mean square error of approximation (RMSEA): < 0.06; and the weighted root mean square residual (WRMR): > 1 [33].

The CSPS-It reliability assessment was performed by testing its internal consistency and stability over time. The internal consistency was examined using Cronbach’s alpha coefficient, with values ≥ 0.70 indicating an acceptable reliability [34]. In addition, the corrected item-total correlation was employed to evaluate the reliability of each item. Values ≥ 0.30 were considered acceptable [35]. The scale stability over time was evaluated using the intra-class correlation coefficient (ICC) of test-retest measures conducted in a sample of 50 nurses after two weeks. These nurses met the same inclusion criteria of other participants. An ICC > 0.70 indicated an acceptable stability [34].

In addition, hypothesis testing was performed to confirm a greater mean compliance among nurses who had participated in at least one training course on SPs compared to others, as supported by several previous studies [15,21,36,37]. T-test was employed for this purpose. Statistical analyses were performed using SPSS 22.0 (IBM Corp., Armonk, NY, USA) and Mplus 7.1 [32].

## 3. Results

### 3.1. Phase 1

#### 3.1.1. Translation and Cross-Cultural Adaptation Process

The cross-cultural adaptation process showed a good adaptability of the original scale in the Italian contest. One important issue that emerged during the adaptation process was the Italian term used for the translation of “*surgical mask*” in the item 13. During the generation of the pre-final version of the CSPS-It, the panel of infection control experts reported that this term was confusing because in Italy “*surgical mask*” usually refers only to medical devices without certification as PPE. This is used to help prevent contamination of the work environment or a sterile field from large particles generated by the wearer/worker and not as PPE. We decided not to translate the term “*surgical*” and instead use the term “*mask*” alone: in Italian this is commonly used to refer to the original meaning of the CSPS item. After this clarification, the CSPS-It was pilot tested.

#### 3.1.2. Content and Face Validity of CSPS-It

The content validity assessment conducted by the panel of experts showed an I-CVI = 0.83 for items 7, 13, 14, 15, 16, and 19. All other items obtained universal agreement (I-CVI = 1). The computed S-CVI was 0.95. During the face validity evaluation of the CSPS-It, participants did not report any language problem or difficulty in understanding and answering the questionnaire. Thus, the final version of the scale was considered ready to be administered for psychometric testing (phase 2).

### 3.2. Phase 2

#### 3.2.1. Demographic Characteristics of the Sample

The sample included 253 registered nurses out of 308 clinical nurses invited to participate in phase 2 of the study. The response rate was 78.9%. The majority of the sample was female (85.4%) and their ages ranged from 26 to 35 years. About 46% of the sample completed one or more first-level professional master program, and 11% of participants had a master’s degree or a higher education level. More than 50% of participants had between 3 and 10 years of job experience. The most represented clinical setting was the adult medical-surgical wards (56.5%). More than 85% of participants had participated in at least one training course on SPs. Further details of participants’ characteristics are presented in Table 1.

#### 3.2.2. Confirmatory Factor Analysis

**Figure 2:**
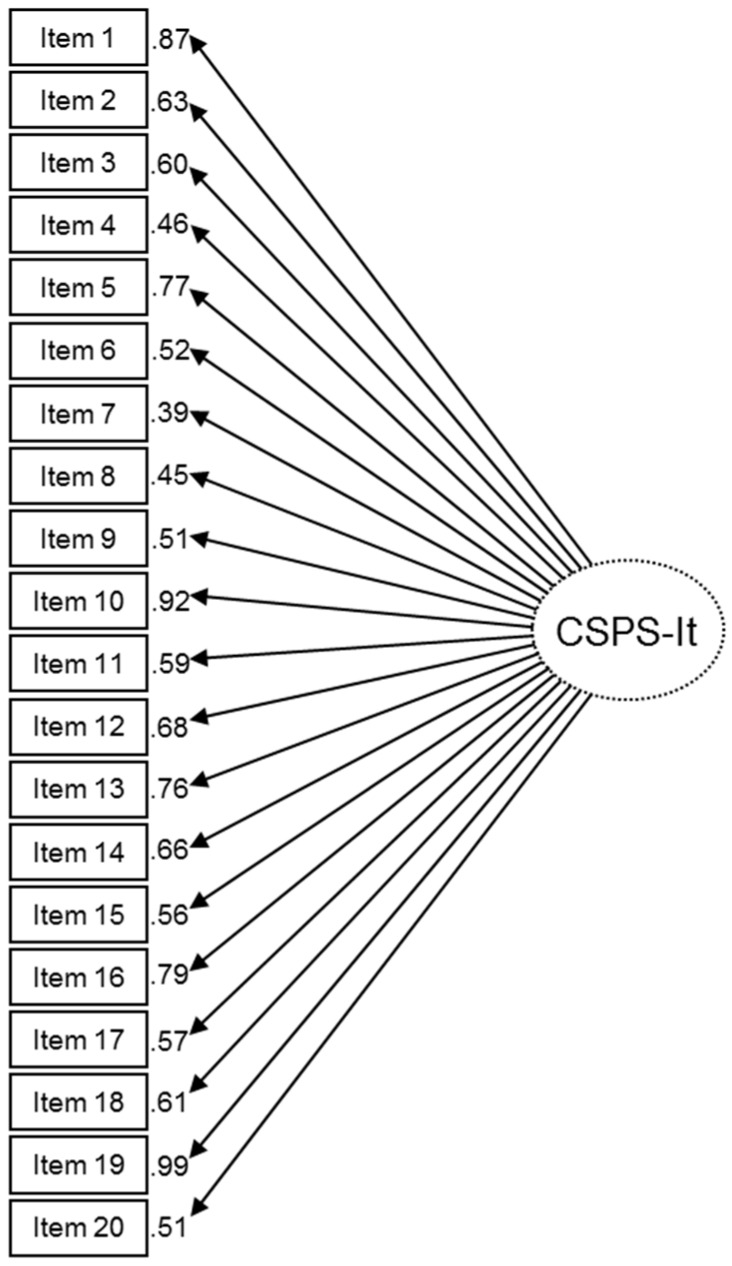
Confirmatory factor analysis (CFA) of the Compliance with Standard Precaution Scale–Italian version (CSPS-It).

In order to assess construct validity, a unidimensional model was tested in line with the practice to compute an overall score [24,25,26,38]. This model yielded acceptable fit indices: χ^2^ = 508.26 (*p* < 0.001), CFI = 0.90, TLI = 0.87, RMSEA = 0.09 (CI 90% = 0.08–0.10), and WRMR = 1.596. Thus, the CSPS-It was considered unidimensional (Figure 2). All the factor loadings were significant (*p* < 0.001) and were ≥ 0.39. In particular, the factor loadings of several items were > 0.75 (items 1, 5, 10, 13, 16, 19). 

#### 3.2.3. Hypothesis Testing

In addition, we conducted hypothesis testing to confirm higher compliance based on participation in at least one training course on SPs. As expected, the compliance of nurses who participated in at least one training course on SPs (mean 14.66, SD = 3.08) was significantly (*p* < 0.001) higher than that reported by those who had never participated in such training (mean = 9.92, SD = 3.94), as shown in Table 2.

#### 3.2.4. Reliability of CSPS-It

The computed Cronbach’s alpha coefficient of the overall scale was 0.84. The computed ITCs ranged from 0.311 to 0.608, as shown in Table 3. The intra-class correlation coefficient for the two-week test-retest of the total scores was 0.86.

#### 3.2.5. Scores

Table 3 presents the scores for each item of the CSPS-It. The compliance rate for the items varied from 36.8% to 93.3%. Five items (5,10,11,14,19) showed a ceiling effect because the endorsement in the upper extreme responses was >80% [31]. The overall compliance rate was 69.9%. The mean of the total score was 13.98 (SD = 4.16; range = 3–20; skewness = −0.44; kurtosis = −0.63).

## 4. Discussion

This study focused on the psychometric evaluation of the CSPS-It among Italian clinical nurses. Our findings showed good evidence supporting the CSPS-It validity and reliability. The cultural adaptation process showed a good applicability of the CSPS-It in the Italian context after a rigorous procedure of translation and evaluation of conceptual equivalence with the CSPS original version. Thus, we confirm the relevance and applicability of the CSPS, as reported in previous studies [9,25,26].

The CSPS-It is able to accurately measure compliance with SPs, as it showed good content, face, and construct validity. The results of the content validity showed optimal ability of all the items to reflect the domain of interest and the conceptual definition of SPs [39]. The result of the face validity of CSPS-It ensured that the instrument is understandable and relevant for the target population [39].

The construct validity of the scale was also successfully tested using CFA and hypothesis testing. This means that the CSPS-It is related to the specified SP behaviours, in accordance with established World Health Organization and the Centers for Disease Control guidelines [24]. It is worth noting that in practice the original version of the scale was considered unidimensional, as a total score was computed [9,24]. However, to the extent of our knowledge, this unidimensionality was never tested in previous studies [9,24,25,26]. The CFA of the CSPS-It showed acceptable fit indices attesting to a unidimensional model of the scale. This could support the use of a total score for assessing compliance with SPs.

In addition, construct validity was confirmed by hypothesis testing. In line with our hypothesis based on previous studies [15,21,36,37], nurses who have participated in at least one training course on SPs showed a higher compliance than nurses who have never participated in such training. This finding highlights the importance of training programs on SPs guidelines for increasing knowledge, skills, and safe behaviours of nurses and confirms the ability of the scale to distinguish between different known groups.

Regarding the reliability of the scale, findings showed a good internal consistency of the scale. Moreover, to further support the reliability of the scale, we found acceptable ITCs for all the items. Finally, a high ICC of the two-week test-retest scores of the CSPS-It revealed its good stability over time to measure the compliance with the SPs of Italian nurses.

The present study does have a few limitations. We have conducted the study in a single centre. This limits the generalizability of the findings. To increase the weight of evidence, a larger sample size from different centers is recommended in order to further improve fit indices at CFA and thereby support the dimensionality of the scale. Moreover, data from this study could have been affected by social desirability bias, in particular for those items showing a ceiling effect. In addition, the questionnaire was administered within an institutional survey and, although anonymous, this could have interfered with the participants’ answers.

Nevertheless, the overall compliance rate shown by the sample underlines several margins of improvement but was higher than the overall compliance rates shown by nurses in Hong Kong, Brazil, and Saudi Arabia in previous studies with the same instrument [8,25]. Compared to other studies that reported participant characteristics, our sample had a similar sex distribution, was younger and less experienced than Brazilian but older and more experienced than Hong Kong nurses [8]. These scholars did not report the nurses’ educational level or if participants received specific training on SPs [8,25]. We found a high educational level in our sample and most of nurses received a SPs training. This could be one of the reasons why the compliance rate of our sample was higher than that found in previous studies [8,25]. However further research is needed to better explain determinants of nurse compliance with SPs across countries.

This study has significant implications for nursing practice and education because the CSPS-It was found valid and reliable, as well as easy to administer and interpret. Moreover, CSPS-It is the first instrument available in Italian to measure compliance with SPs. This can foster the planning and evaluation of new interventions in order to increase and ensure stringent compliance with SPs.

## 5. Conclusions

SPs represent the most important infection prevention and control actions to reduce transmission of microorganisms to other patients or to healthcare providers [5]. Thus, supporting nurses to engage in such behaviours, as first line workers, is a core component of infection control. Managers should pay greater attention in monitoring compliance with SPs among clinical nurses in order to implement and evaluate interventions relevant to their needs.

The validity and reliability assessment of CSPS-It showed satisfactory results. This version will allow the conduction of further studies about compliance with SPs in Italy and will make intercultural comparisons and collaboration possible in favor of progress in this specific field of research.

## Figures and Tables

**Table 1:** Demographic characteristics of the sample (n = 253).

Variable	n	%
Sex		
Male	37	14.6
Female	216	85.4
Age		
<25	24	9.5
26–30	71	28.1
31–35	76	30.0
36–40	46	18.2
>40	36	14.2
Education		
Degree	118	46.6
Master	106	41.9
Master’s degree	29	11.5
Clinical experience (years)		
<3	36	14.2
3–6	51	20.2
7–10	84	33.2
11–14	36	14.2
>14	46	18.2
Clinical setting		
Adult medical-surgical	143	56.5
Intensive care unit	19	7.5
Operation theater	44	17.4
Outpatient units	47	18.6
SPs training *		
Yes	217	85.8
No	36	14.2

* Participation at least in one training course on standard precautions (SPs).

**Table 2:** Hypothesis testing for compliance with SPs (CSPS-It total score).

Variable	Mean (SD)	*p*
SPs training *		
Yes	14.66 (3.08)	<0.001
No	9.92 (3.94)	

* Participation at least in one training course on SPs

**Table 3:** Compliance rate and item total correlation coefficients of the CSPS-It.

Item	Frequency of Endorsement, %				Compliance Rate %	Corrected Item-Total Correlation
	Never	Seldom	Sometimes	Always		
1. I wash my hands between patient contacts	0.0	0.0	22.5	77.5	77.5	0.608
2. I only use water for hand washing	74.3	19.8	5.9	0.00	74.3	0.444
3. I use alcohol hand rubs as an alternative if my hands are not visibly soiled	0.0	0.8	33.6	65.6	65.6	0.426
4. I recap used needles after giving an injection	77.1	19.8	3.2	0.0	77.1	0.323
5. I put used sharp articles into sharps boxes	0.0	0.0	6.7	93.3	93.3	0.439
6. The sharps box is only disposed when it is full	46.6	49.4	2.4	1.6	46.6	0.404
7. I remove PPE in a designated area	0.0	1.6	32.8	65.6	65.6	0.311
8. I take a shower in case of extensive splashing even after I have put on PPE	0.8	4.7	57.7	36.8	36.8	0.314
9. I cover my wound(s) or lesion(s) with waterproof dressing before patient contacts	0.0	0.4	32.4	67.2	67.2	0.320
10. I wear gloves when I am exposed to body fluids, blood products, and any excretion of patients	0.0	0.4	7.9	91.7	91.7	0.385
11. I change gloves between each patient contact	0.0	0.0	11.1	88.9	88.9	0.364
12. I decontaminate my hands immediately after removal of gloves	0.0	1.2	33.6	65.2	65.2	0.495
13. I wear a surgical mask alone or in combination with goggles, face shield, and apron whenever there is a possibility of a splash or splatter	0.0	1.6	39.5	58.9	58.9	0.516
14. My mouth and nose are covered when I wear a mask	0.0	0.4	7.1	92.5	92.5	0.342
15. I reuse mask or disposable PPE	71.9	26.1	2.0	0.0	71.9	0.422
16. I wear a gown or apron when exposed to blood, body fluids, or any patient excretions	0.0	1.6	45.5	53.0	53.0	0.580
17. Waste contaminated with blood, body fluids, secretion, and excretion are placed in red plastic bags irrespective of patient’s infective status	0.4	1.6	38.7	59.3	59.3	0.443
18. I decontaminate surfaces and equipment after use	1.2	2.4	40.3	56.1	56.1	0.498
19. I wear gloves to decontaminate used equipment with visible soils	0.0	0.0	7.9	92.1	92.1	0.443
20. I clean up spillage of blood or other body fluid immediately with disinfectants	1.2	4.0	30.0	64.8	64.8	0.391
